# Effects of eccentric training on athletes’ explosive power: a systematic review and meta-analysis

**DOI:** 10.3389/fphys.2026.1960414

**Published:** 2026-10-12

**Authors:** Yugang Zhang, Lei Zhang, Baozhong Zhang, Min Sun, Yuan Zhuang, Ni Ma

**Affiliations:** 1 Department of Physical Education, Yuncheng University, Yuncheng, China; 2 College of Sport and Health Science, Chongqing Normal University, Chongqing, China; 3 School of Physical Education, Baoshan University, Baoshan, Yunnan, China; 4 Institute of Physical Education, Northwest Minzu University, Lanzhou, Gansu, China

**Keywords:** athletes, eccentric training, meta-analysis, power performance, training duration

## Abstract

**Background:**

Eccentric training is frequently used to improve explosive performance in athletes; however, findings across different performance dimensions (e.g., jumping and sprinting) remain inconsistent, and intervention heterogeneity is substantial. This study aimed to systematically evaluate the effects of eccentric training on explosive performance in athletes.

**Methods:**

Web of Science, PubMed, CNKI, Wanfang, Scopus, and SPORTDiscus were systematically searched. Randomized controlled trials (RCTs) were included according to the PICOS framework (healthy athletes; explicitly labeled eccentric training; conventional training as control; outcomes related to explosive performance, including countermovement jump (CMJ) and linear sprint performance over 10 m, 20 m, and 30 m). Only studies explicitly labeled as eccentric training were included; Nordic hamstring exercise and other interventions not explicitly labeled as eccentric training were excluded. Methodological quality was assessed using the Physiotherapy Evidence Database (PEDro) scale, and meta-analyses were performed using a random-effects model.

**Results:**

Eleven RCTs (n = 348; 11–25 years; 6–24 weeks) were included. Pooled results showed that eccentric training significantly improved CMJ performance (MD = 2.92 cm, *p* = 0.0006). However, no significant improvements were observed for short-distance sprint performance: 10-m and 20-m sprint (MD = −0.10 s, *p* = 0.11) and 30-m sprint (MD = −0.42 s, *p* = 0.23). The 10-m sprint meta-analysis showed high heterogeneity, with two studies reporting significant effects and two reporting non-significant effects. Subgroup analysis indicated that eccentric training duration had a moderating effect on CMJ performance (MD = 1.91 cm, p = 0.009).

**Conclusion:**

Eccentric training may improve CMJ, a component of jump performance, in athletes, but shows no significant effect on linear sprint performance (20 m and 30 m), with unstable evidence for 10 m. Due to the limited number of included studies, substantial heterogeneity, moderate-to-high risk of bias, and inclusion of only studies explicitly labeled as eccentric training, these conclusions should be interpreted cautiously and cannot be generalized to other explosive performance dimensions or all eccentric training modalities. Future high-quality studies are needed to standardize eccentric training protocols, intervention durations, and outcome measurements, and to perform stratified analyses by outcome dimension.

**Systematic Review Registration:**

https://www.crd.york.ac.uk/prospero/display_record.php?RecordID=1380375, identifier CRD420261380375.

## Introduction

Athletes’ competitive performance in sport is highly dependent on the physical reserves and athletic capacity acquired through long-term systematic training (Siramaneerat and Chaowilai, 2022). Systematic evaluation and diagnosis of the effects of acquired training can help coaches clearly understand the physiological adaptation mechanisms of a given training program and provide empirical evidence for optimizing training strategies (Den Hartigh et al., 2018). Among the many physical factors influencing athletes, explosive power is an important indicator reflecting rapid neuromuscular force-generating capacity and a key determinant of multi-joint synergistic force production efficiency and power output during high-intensity exercise (Aksović et al., 2020). For example, key technical actions such as the drive and turn in shot put and the whip-like braking in javelin throwing depend heavily on efficient coordinated force transmission among the upper limbs, trunk, and lower limbs; the quality of an athlete’s explosive power directly affects release velocity and athletic performance (Takanashi et al., 2022). Similarly, in baseball, training aimed at improving explosive power has been shown to enhance rate of force development and kinetic chain efficiency, thereby influencing throwing and batting power performance (Aydin et al., 2015). It should be noted that this review treats “explosive performance” as a multidimensional construct, including power output, rate of force development (RFD), jump performance, sprint performance, change-of-direction performance, and sport-specific explosive actions. These dimensions reflect distinct neuromuscular determinants and therefore cannot be simply pooled into a single “explosive power” outcome. In this study, CMJ and SJ were classified as jump performance, while 10 m, 20 m, and 30 m were classified as sprint performance; each indicator was extracted and pooled separately by dimension. Accordingly, identifying training methods that improve explosive performance in athletes and thereby enhance competitive performance has become an important issue in training practice.

In daily training, coaches attempt to improve athletes’ explosive power through various training methods, such as unilateral training (Ramírez-Campillo et al., 2015), resistance training (Santos and Janeira, 2012), high-intensity interval training (Fajrin and Kusnanik, 2018), and eccentric training (Xiong, 2022). Among these, eccentric training is a strength training method in which the muscle is lengthened by an external force while actively contracting. By enhancing muscle tension and neuromuscular adaptation, it improves muscle injury resistance and explosive power output (Zhang et al., 2024), and it has also been applied in sports rehabilitation (Liu, 2019).

However, eccentric training is not a single training modality. Broadly, it includes flywheel training, eccentric squat, Achilles tendon drop, and Nordic hamstring exercise, among others, and these forms differ fundamentally in involved muscle groups, joint loading patterns, and eccentric overload characteristics. It should be noted that this review only included studies explicitly labeled as “eccentric training” and did not include interventions such as Nordic hamstring exercise that were not explicitly labeled as eccentric training. Nevertheless, the included studies still varied considerably in training equipment, movement form, eccentric load, contraction velocity, frequency, duration, and concentric coupling. Coaches often use eccentric training methods such as eccentric squat to improve athletes’ jumping ability (Li, 2023), agility (Wang et al., 2024), and muscle strength (Bai et al., 2025). These performance improvements are closely related to improvements in explosive performance (Zamparo et al., 2002). From a biological mechanism perspective, eccentric training optimizes the efficiency of energy storage and release during the muscle stretch-shortening cycle (SSC), enhances neuromuscular coordination, and thereby promotes explosive power development. At the practical level, the positive intervention effects of eccentric training on explosive power-dominant athletic performance have also been empirically tested (Hou and Wang, 2023). Therefore, clarifying the logical relationship between eccentric training, improvements in explosive performance, and enhanced athletic performance has important theoretical and applied value for optimizing training practice.

However, existing studies on eccentric training are mostly conducted in specific sports with small sample sizes, and training intensity and frequency parameters vary, leading to some discrepancies in findings. More importantly, because different eccentric training interventions involve different biomechanical mechanisms, their differential effects on various dimensions of explosive performance (sprint, jump, and change of direction) have not been systematically quantified. In addition, current quantitative reviews on eccentric training mostly focus on sports rehabilitation (Stone et al., 2023) or athletic performance (Tsartsapakis et al., 2026). No study has systematically compared, while distinguishing specific implementation modes of eccentric training, the net effect sizes of eccentric training versus conventional training on various dimensions of explosive performance (e.g., jump height, sprint speed, throwing/batting power). Given the significant heterogeneity in eccentric training interventions regarding modality, load, frequency, duration, and concentric coupling, this study will conduct subgroup analyses by training modality and outcome dimension; if data are insufficient, a qualitative synthesis will be performed with explicit acknowledgment of the sources of heterogeneity and their impact on conclusions. Accordingly, this study employs meta-analysis to systematically integrate existing empirical literature, aiming to quantitatively evaluate the effects of eccentric training on various dimensions of explosive performance in athletes, in order to provide high-level evidence for coaches to develop scientific training programs.

## Methodology

### Protocol and registration

The present systematic review and meta-analysis adheres to the PRISMA guidelines (Page et al., 2021), and its protocol was prospectively registered with PROSPERO (registration no. CRD420261380375).

### Eligibility criteria

The eligibility criteria for the present study were developed in accordance with the PICOS framework (Population, Intervention, Comparison, Outcomes, and Study Design; see **Table 1**). Only studies investigating the effects of eccentric training on explosive performance in athletes were deemed eligible. Consequently, studies fulfilling the following criteria were included:

**Table 1:** Inclusion criteria based on the PICOS framework.

Item	Inclusion criteria
Population	Athletes (male/female)
Intervention	Eccentric training
Comparison	Traditional training
Outcome	Vertical jump performance: countermovement jump (CMJ) height (cm); Linear sprint performance: 10-m, 20-m, and 30-m sprint time (s); Agility: e.g., T-test time (s); Change-of-direction: e.g., 505 test time (s).
Study designs	Randomized controlled trial (RCT)

1. Peer-reviewed studies published in either Chinese or English were included. Participants were male or female athletes, and the studies were designed to investigate the effects of eccentric training on athletes’ explosive power. Eligible studies were required to adopt a randomized controlled trial (RCT) design with at least two groups.
2. Included studies were required to implement a planned, structured eccentric training intervention aimed at improving athletes’ explosive performance. This review only included studies explicitly labeled as “eccentric training”; interventions such as Nordic hamstring exercise that were not explicitly labeled as eccentric training were excluded. Eccentric training was defined as a strength training method in which the muscle is lengthened by an external force while actively contracting. In addition, studies combining eccentric training with other training methods (e.g., sport-specific skill training) were also considered eligible.
3. Included studies were required to examine the effects of eccentric training on athletes’ explosive performance and to report at least one explosive performance-related outcome measure (e.g., countermovement jump, short-distance sprint). In this study, explosive performance was treated as a multidimensional construct, including jump performance (e.g., countermovement jump, squat jump) and sprint performance (e.g., 10 m, 20 m, 30 m). Each outcome was extracted and pooled separately by dimension.
4. No restrictions were imposed on the sport type, age, or sex of the athletes. Furthermore, no limitations were placed on sample size, study location, or intervention duration.

1. Studies that included non-training interventions (e.g., psychological interventions) in addition to eccentric training, as well as those without supervised training sessions, were excluded.
2. Studies published in languages other than Chinese or English, and dissertations and conference proceedings, were excluded.
3. Studies that did not adopt an RCT design were excluded.

### Search strategy and selection process

On July 20, 2026, we searched Web of Science, PubMed, China National Knowledge Infrastructure (CNKI), Wanfang Data, Scopus, and SPORTDiscus separately. To ensure comprehensive identification of eligible studies, the search strategy was developed based on previous studies (Zhang et al., 2024; Zhuang et al., 2026) and incorporated subject heading mapping using database thesauri (e.g., Medical Subject Headings [MeSH]), synonym expansion, and consultations with subject experts and librarians (see **Supplementary Table S1** for details). For the literature searches in the six databases, the following search terms and operators were used: (eccentric training OR flywheel overload training OR eccentric overload OR flywheel exercise OR eccentric overload performance OR flywheel sport OR Yo-Yo sport OR Yo-Yo exercise OR Yo-Yo training) AND (explosive power OR explosive strength OR power output OR rate of force development OR jump performance OR sprint performance OR plyometric performance) AND (randomised controlled trials OR randomised controlled trial). To identify further relevant studies, we screened the reference lists of published systematic reviews and included studies. Finally, to ensure the accuracy of the search process, a librarian was consulted to support data collection and searching.

The study selection process consisted of five stages (**Figure 1**). In the first stage, duplicate records were removed. In the second stage, records were excluded after title and abstract screening if they did not involve athletes, were review articles, or were not relevant to the research topic. In the third stage, dissertations and conference proceedings were excluded after title and abstract screening, and studies of poor methodological quality were excluded during quality assessment. In the fourth stage, the full texts of the remaining articles were thoroughly read to determine final eligibility. In the fifth stage, studies were ultimately selected for inclusion based on the eligibility criteria and quality assessment. Throughout the screening process, two investigators (ZYG and SM) worked independently; any disagreements were resolved through discussion with a third investigator (ZL) until consensus was reached. After this final screening, 12 studies were included in the meta-analysis.

**Figure 1:**
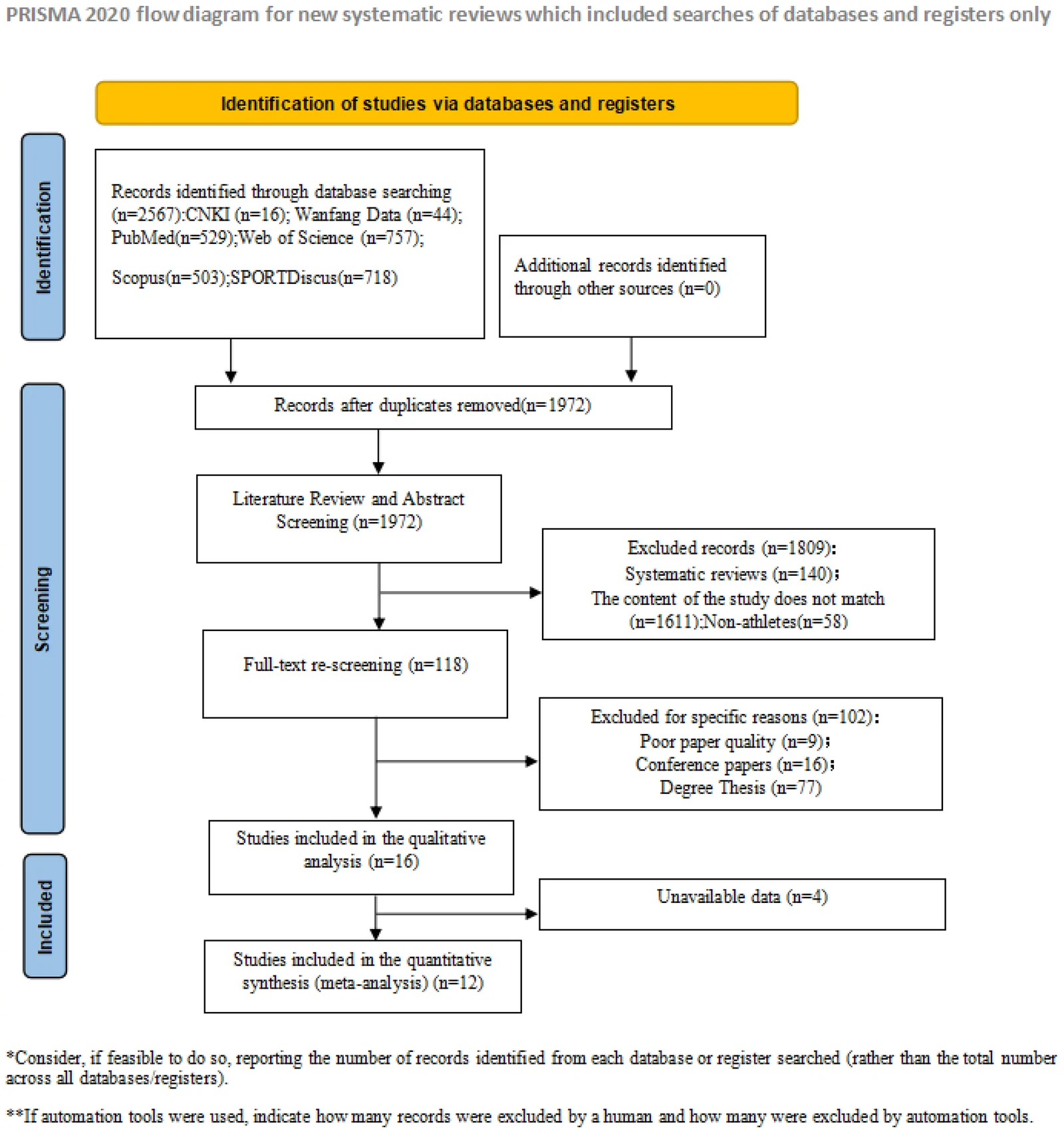
PRISMA flow diagram.

### Data extraction

Data extraction was performed by two authors (ZYG and SM) using a Microsoft Excel spreadsheet, and the extracted data were cross-checked by a third author (ZL). Any disagreements were resolved through discussion among all three authors. The following data were extracted: (a) basic study information (first author, year of publication, and country); (b) participant characteristics (age, sport, sex, training experience, competitive level, and sample size); (c) detailed information on the eccentric training intervention, including duration, frequency, and training type, as well as equipment, movement form, eccentric load, contraction velocity, and whether it was coupled with concentric training; (d) outcome assessment: after consensus among all authors, multiple explosive performance-related technical indicators were extracted, including but not limited to jump performance (e.g., countermovement jump, squat jump) and sprint performance (e.g., 10 m, 20 m, and 30 m short-distance linear sprint time). Each outcome was extracted separately by dimension, and jump and sprint outcomes were not pooled into a single “explosive power” endpoint; (e) pre- and post-intervention means and standard deviations (SDs) for outcome measures in the experimental and control groups. If means and SDs were not reported in the included studies, they were calculated according to the formulas recommended by the Cochrane Handbook using confidence intervals (CIs), standard errors (SEs), or t-values. When extracting variables, characteristics such as training modality, sport, and competitive level were also recorded to assess clinical heterogeneity. (f) Management of unreported data: if any of the above items were not explicitly reported in the original study, they were coded as “not reported (NR)” in our extraction spreadsheet.

### Quality assessment and risk of bias

Methodological quality and risk of bias of the included studies were assessed using the PEDro scale and the Cochrane Risk of Bias tool. The combined use of these two instruments for comprehensive evaluation has been widely adopted in the literature (de Morton, 2009; Sun et al., 2025). Two reviewers (ZYG and SM) independently evaluated methodological quality using the PEDro scale. The results were then cross-checked by a third author (ZL), and any disagreements were resolved through discussion until consensus was reached among the three authors. The PEDro scale comprises 11 items related to methodological quality; each criterion satisfied scores 1 point, yielding a total score of 10 (item 1, which addresses external validity, is not included in the total score). Scores range from 1 to 10, with ≤3 indicating poor quality, 4–5 fair quality, and 6–10 high quality. To reduce the risk of bias in the included studies, trials with a PEDro score of ≤3 were excluded. In addition, two reviewers (ZY and ZBZ) assessed risk of bias using RevMan 5.4.1 software in accordance with the Cochrane Handbook guidelines. For each domain, the risk of bias was categorized as “low risk,” “unclear risk,” or “high risk.” An overall risk-of-bias judgment was then determined for each study (**Figure 2**). In summary, four independent reviewers—ZYG, SM, ZY, and ZBZ—performed the evaluations using the PEDro scale and RevMan tool, respectively; when disagreements arose between reviewers, a third reviewer was consulted to resolve them.

**Figure 2:**
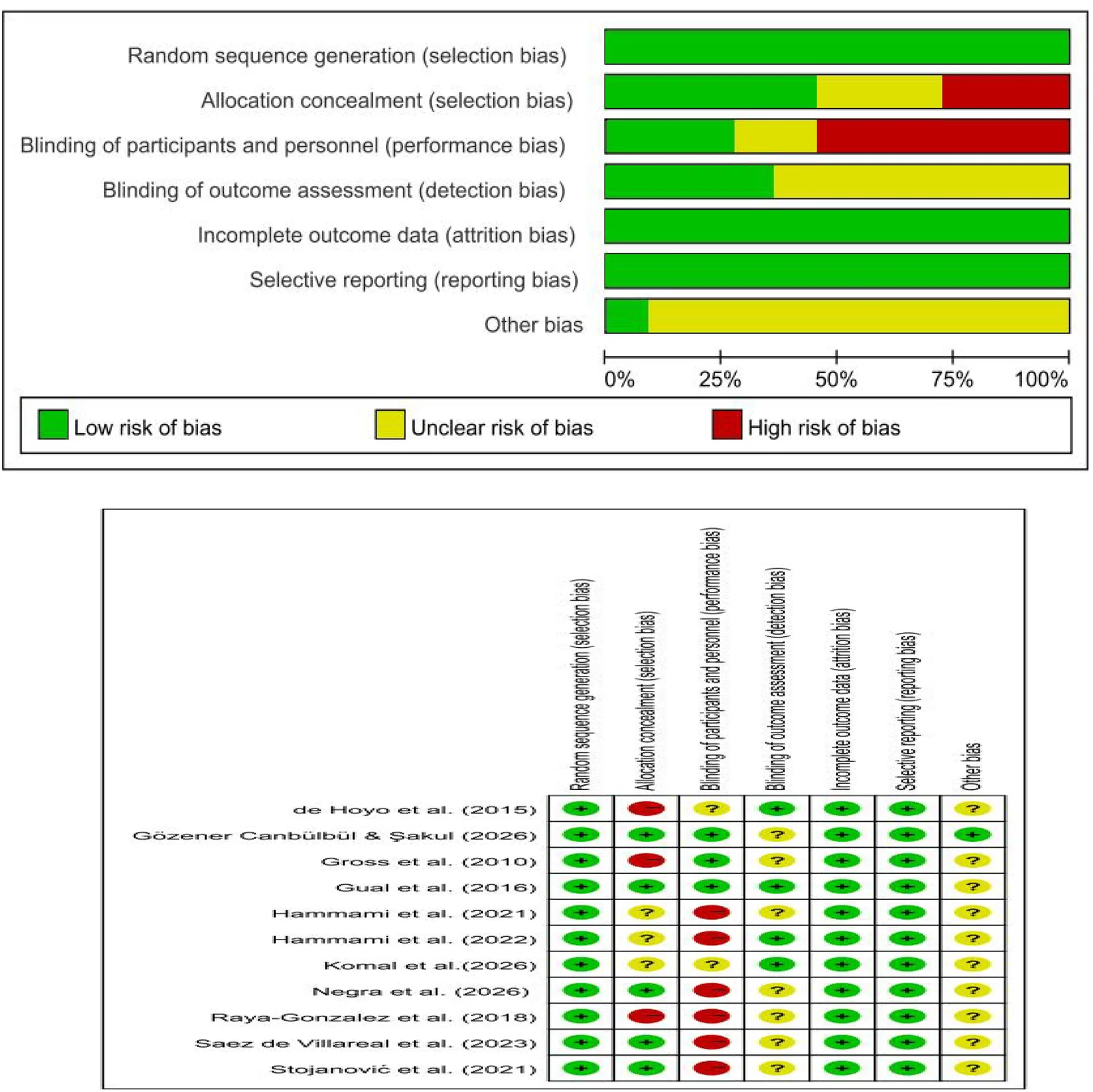
Risk of bias assessment.

### Statistical analysis

Effect sizes were calculated as mean differences (MD) with 95% confidence intervals. The effect size for each outcome was derived from pre- and post-intervention means and standard deviations. Reporting MD in original units provides an intuitive reflection of the actual intervention effect, facilitating practical application and clinical interpretation.

If means and standard deviations were not directly reported in the original articles, corresponding authors or first authors were contacted via email to request **Supplementary Data**. For studies presenting results in graphical format, data were extracted from the figures using WebPlotDigitizer (version 4.5; https://apps.automeris.io/wpd/) (Drevon et al., 2016).

Meta-analyses were conducted only when at least three independent studies provided data for a given outcome measure. Although statistical models can accommodate as few as two studies (Valentine et al., 2010), given the typically small sample sizes common in sport science research, we required three or more studies per outcome to ensure the robustness of the pooled estimates (Deng et al., 2023). In this review, explosive performance was treated as a multidimensional construct. Outcomes were classified into jump performance (CMJ and SJ) and sprint performance (10 m, 20 m, and 30 m). Meta-analyses were performed separately within each outcome domain; jump and sprint outcomes were not pooled into a single “explosive power” endpoint. When multiple indicators within the same domain differed in units or direction, they were analyzed and reported separately or standardized before pooling where appropriate.

Pooled effect sizes were calculated using the inverse-variance random-effects model (Deeks et al., 2019). This model accounts for both within-study sampling error and between-study heterogeneity, which is particularly relevant given the anticipated clinical and methodological diversity across studies (e.g., variations in participant age ranges, training protocols, and outcome measurement tools). Compared to the fixed-effect model, this approach provides a more conservative and generalizable pooled estimate. It should be noted that we applied the random-effects model uniformly across all meta-analyses and did not use the fixed-effect model for any outcome. Furthermore, because the random-effects model incorporates between-study variance into the standard error of the pooled estimate and remains valid even in the presence of high heterogeneity *(I²* > 75%), heterogeneity was not used as an exclusion criterion for conducting meta-analyses.

It is important to note that because the pooled effect sizes are reported as MDs in their original units, these values do not permit a uniform classification of small, medium, or large effects using conventional thresholds for standardized effect sizes. The statistical significance of the pooled effects was therefore determined primarily by the P-value (< 0.05) and whether the 95% confidence interval contained zero. In cases where a study included multiple intervention arms, the control group was proportionally split across the relevant comparisons to avoid unit-of-analysis errors (Deeks et al., 2019).

Between-study heterogeneity was assessed using the I² statistic, with the following thresholds: <25% indicating low heterogeneity, 25%–75% moderate heterogeneity, and >75% high heterogeneity (Higgins and Thompson, 2002). As stated above, the I² statistic was used solely to quantify the degree of heterogeneity and to inform sensitivity analyses and interpretation of the results, rather than as a threshold criterion for meta-analysis. Given the substantial heterogeneity in eccentric training interventions (e.g., equipment, movement form, eccentric load, contraction velocity, frequency, duration, and concentric coupling), I² was also used to guide subgroup and sensitivity analyses. When heterogeneity remained high and could not be explained, pooling was not forced, and a structured narrative synthesis was used instead.

Moderator variable analysis strategy: Training intensity, sport type, and competitive level were pre-specified as potential moderators for inclusion in subgroup analyses or meta-regression models to explain the differential effects of various eccentric training modalities across different athlete populations. Eccentric training intervention characteristics—including equipment, movement form, eccentric load, contraction velocity, frequency, duration, and concentric coupling—were also considered as potential sources of heterogeneity. Subgroup analyses by eccentric training modality/characteristics and outcome domain were planned. If data were insufficient for quantitative subgroup analysis, findings were summarized qualitatively, and the sources of heterogeneity and their potential influence on conclusions were explicitly reported. The specific analytical framework was as follows: (a) age and training duration were included in meta-regression (as continuous variables) and subgroup analyses (age: <18 years vs. ≥18 years; duration: <8 weeks vs. ≥8 weeks); (b) training intensity and competitive level were examined via subgroup analyses (competitive level: professional/elite vs. amateur/collegiate vs. unreported). However, given the anticipated inconsistency in reporting standards and the likely high proportion of missing data in the original studies, these analyses were intended only for qualitative trend exploration and were not incorporated into the quantitative models.

Sensitivity analysis strategy: (a) A leave-one-out sensitivity analysis was performed to identify studies with extreme influence. Sensitivity analyses also considered the influence of eccentric training modality and outcome domain where data permitted. (b) Because no outcome included ≥10 studies, meta-regression for continuous variables (e.g., age, training duration) was not performed.

Publication bias was not formally assessed because fewer than 10 studies were included for each outcome. Egger’s linear regression test and trim-and-fill analysis were therefore not performed, and funnel plots were not used as evidence of robustness (Sedgwick, 2013). Due to the limited number of studies per outcome, planned subgroup analyses by eccentric training characteristics and outcome domains could not always be performed; this is acknowledged as a limitation. This limitation should be considered when interpreting the pooled estimates.

All statistical analyses were performed using Comprehensive Meta-Analysis software (version 4.0; Biostat, Inc., Englewood, NJ, USA), and forest plots were generated using Review Manager 5.4. The threshold for statistical significance was set at *p* < 0.05.

## Results

### Study selection

As shown in **Figure 1**, an initial database search identified 2,567 records. After duplicates were removed, 1,927 records remained. Title and abstract screening of these records identified 118 potentially eligible articles for full-text review. After full-text review, 102 articles were excluded, and 16 articles met the inclusion criteria. Finally, during data extraction, four articles were excluded because the required specific information was unavailable; ultimately, 12 articles were included in the meta-analysis.

### Study quality assessment and risk of bias

The methodological quality of the included studies was assessed using the PEDro scale. The results showed that three studies scored 7, indicating relatively high quality, and eight studies scored 4 to 5, indicating moderate methodological quality. However, one study (Sabido et al., 2017) received a score of 3 or less and was therefore excluded from this review (see **Table 2**).

**Table 2:** Physiotherapy evidence database (PEDro) scale ratings.

Study name	N1	N2	N3	N4	N5	N6	N7	N8	N9	N10	N11	Total*	Study quality
Gözener Canbülbül and Şakul (2026)	1	1	1	1	0	0	1	1	0	1	1	7	H
de Hoyo et al. (2015)	1	0	0	1	0	0	0	1	0	1	1	4	M
Sáez de Villareal et al. (2023)	1	1	0	1	0	0	0	1	0	1	1	5	M
Gross et al. (2010)	1	1	0	1	0	0	0	0	0	1	1	4	M
Gual et al. (2016)	1	1	1	1	0	0	1	1	0	1	1	7	H
Hammami et al. (2022)	1	1	0	1	0	0	0	0	0	1	1	4	M
Hammami et al. (2021)	1	1	0	1	0	0	1	1	1	1	1	7	H
Komal et al. (2026)	1	0	0	1	0	0	1	1	0	1	1	5	M
Negra et al. (2026)	1	1	0	1	0	0	0	1	0	1	1	5	M
Raya-Gonzalez et al. (2018)	1	1	0	1	0	0	0	1	0	1	1	5	M
Sabido et al. (2017)	1	0	0	1	0	0	0	0	0	1	1	3	L
Stojanović et al. (2021)	1	1	0	1	0	0	0	1	0	1	1	5	M

A detailed explanation for each PEDro scale item can be accessed at https://www.pedro.org.au/english/downloads/pedro-scale.

*From a possible maximal punctuation of 10.L, Low; M, Moderate; H, High.

Two reviewers (ZYG and SM) independently assessed the 11 included studies using the Cochrane Risk of Bias tool (RevMan 5.4.1). For each study, the risk of bias across seven domains was judged as “low risk,” “unclear risk,” or “high risk,” and an overall risk-of-bias judgement was made based on the domain ratings (**Figure 2**). The results showed that most studies were rated as low risk for random sequence generation and incomplete outcome data. Performance bias was generally rated as high risk because blinding of participants and personnel was not possible in exercise-based interventions. Allocation concealment and other bias domains were predominantly rated as unclear risk because of insufficient reporting in the original studies. Overall, the included studies were judged to have a moderate-to-high risk of bias; therefore, caution should be exercised when interpreting the pooled results, with particular attention to the potential influence of expectancy effects and detection bias.

### Study characteristics

A total of 11 randomized controlled trials (RCTs) involving 348 athletes (51 female athletes) were included in this review; the athletes were aged 11 to 25 years. **Table 3** presents the participant characteristics and intervention protocols of the included RCTs. The studies were published between 2010 and 2026. Of the included studies, two adopted a three-arm design (Sáez de Villareal et al., 2023; Stojanović et al., 2021), and nine used a two-arm design (Gözener Canbülbül and Şakul, 2026; de Hoyo et al., 2015; Gross et al., 2010; Gual et al., 2016; Hammami et al., 2021, 2022; Komal et al., 2026; Negra et al., 2026; Raya-Gonzalez et al., 2018). Regarding the countries of origin, two studies were conducted in the United Kingdom (Hammami et al., 2021, 2022), four in Spain (de Hoyo et al., 2015; Sáez de Villareal et al., 2023; Gual et al., 2016; Raya-Gonzalez et al., 2018), and one each in Turkey (Gözener Canbülbül and Şakul, 2026), Switzerland (Gross et al., 2010), India (Komal et al., 2026), Germany (Negra et al., 2026), and Serbia (Stojanović et al., 2021). In terms of sports, one study involved volleyball players (Gözener Canbülbül and Şakul, 2026), three involved soccer players (de Hoyo et al., 2015; Negra et al., 2026; Raya-Gonzalez et al., 2018), two involved handball players (Sáez de Villareal et al., 2023; Hammami et al., 2021), one involved skiers (Gross et al., 2010), three involved basketball players (Gual et al., 2016; Komal et al., 2026; Stojanović et al., 2021), and one involved weightlifters (Hammami et al., 2022). The intervention duration ranged from 6 to 24 weeks, with training frequencies of 1–3 sessions per week.

**Table 3:** Characteristics of participants and training interventions in the included studies.

Study	Country	Intervention	N	Sex	Age	Level/ experience	Sport	Comparison	Outcome (s)
Gözener Canbülbül and Şakul (2026)	Türkiye	Freq: 3times/ week Time: 90mins Length: 8weeks	26	F	EG:16.86 ± 0.95 CG:17.58 ± 1.16	Youth academy NR	Volleyball	EG: Traditional training +Eccentric heel-drop training CG: Traditional training	CMJ↑ SJ↑
de Hoyo et al. (2015)	Spain	Freq:1–2 times/week (first 2 weeks:1 time/week; remaining8 weeks:2 times/week) Time: NR Length:10 weeks	33	M	EG:18 ± 1 CG:17 ± 1	Junior elite NR	Football	EG: Traditional training+Eccentric overload training CG: Traditional training	CMJ↑ 10M↑ 20M↑
Sáez de Villareal et al. (2023)	Spain	Freq: 3times/ week Time: 60mins Length: 8weeks	36	M	EG1:19.8 ± 2.2 EG2:20.5 ± 2.5 CG:20.6 ± 1.6	Division≥5 club years ≥5Yrs	Handball	EG1:Plyometric training EG2:Eccentric overload training CG: Traditional training	CMJ↑ 15M→
Gross et al. (2010)	Switzerland	Freq: 3times/ week Time: 60mins Length: 6weeks	15	M	17.6 ± 1.4	National Level Athlete NR	Ski athletes	EG: Traditional strength training+Eccentric cycle ergometer training CG: Traditional strength training	SJ↑ CMJ↑ DJ↑
Gual et al. (2016)	Spain	Freq: 1times/ week Time: 20mins Length: 24weeks	53	26F、27M	CG:24.5 ± 5.5 EG:22.5 ± 3.8	club level NR	Basketball、Volleyball	EG: Eccentric overload squat training CG: Traditional training	CMJ↑
Hammami et al. (2022)	UK	Freq: 2times/ week Time: NR Length: 6weeks	20	M	13.2 ± 0.4	NR	Weightlifters	EG: Eccentric hamstring training CG: Traditional training	10M↑ 30M↑
Hammami et al. (2021)	UK	Freq: 2times/ week Time: NR Length: 6 weeks	45	M	EG1:11.24 ± 0.93 EG1:14.00 ± 0.20 CG1:11.03 ± 0.77 CG2:13.85 ± 0.87	club level NR	Handball	EG: Traditional training +Eccentric training CG: Traditional training	10M↑ 20M↑ 30M↑ T↑
Komal et al. (2026)	India	Freq: 3 times/week Time: NR Length: 6 weeks	32	M	18–25 years	state-level NR	Basketball	EG: Traditional basketball training+Eccentric loading training CG: Traditional basketball training	Vertical Jump ↑
Negra et al. (2026)	Germany	Freq: 2 times/week Time: 15-25mins Length: 8weeks	36	M	EG:12.50 ± 0.24 CG:12.40 ± 0.22	Regional Level 4.0 ± 1.2 years	Soccer	EG: Eccentric training+Traditional football training CG: Traditional football training	CMJ↑ SLJ↑ 20 m↑ COD↑
Raya-Gonzalez et al. (2018)	Spain	Freq: 2 times/week Time: NR Length: 6weeks	16	M	EG:14.7 ± 0.3 CG:14.7 ± 0.2	Elite Junior NR	Soccer	EG: Eccentric overload training+Traditional football training CG: Traditional football training	CMJ↑ 20M↑ 30M↑
Stojanović et al. (2021)	Serbia	Freq:1-2times/week Time: NR Length:8weeks	36	M	EG1:17.58 ± 0.52 EG2:17.52 ± 0.58 CG:17.56 ± 0.54years	regional level EG1:6.17 ± 1.19 EG2:6.92 ± 2.88 CG:6.58 ± 1.38	Basketball	EG1:Eccentric overload training + regular basketball training EG2:Traditional strength training + regular basketball training CG:regular basketball training	CMJ↑ 5 m↑ 20 m↔

F, female; M, male; yrs, years; Freq, frequency; NR, not reported; EG, experimental group; CG, control group; CMJ, countermovement jump; SJ, squat jump; DJ, drop jump; SLJ, standing long jump; COD, change of direction; T, T-test; 5M, 5 meter sprint; 10M, 10 meter sprint; 15M, 15 meter sprint; 20M, 20 meter sprint; 30M, 30 meter sprint.

### Meta-analysis results

#### Effects of eccentric training on vertical jump performance

Of the included studies, eight reported data on countermovement jump (CMJ) performance, involving a total of 227 athletes (de Hoyo et al., 2015; Gözener Canbülbül and Şakul, 2026; Gross et al., 2010; Gual et al., 2016; Negra et al., 2026; Raya-Gonzalez et al., 2018; Sáez de Villareal et al., 2023; Stojanović et al., 2021). The random-effects meta-analysis showed that the eccentric training group achieved significantly greater improvements in CMJ compared with the conventional training group (MD = 2.92 cm, 95% CI:1.26 to 4.59, Z = 3.44, *p* < 0.001). Heterogeneity testing revealed moderate between-study heterogeneity (Tau² = 2.88, Chi² = 14.72, df = 7, *p* = 0.04, *I²* = 52%; **Figure 3**). However, given the limited number of included trials, this heterogeneity estimate should be interpreted with caution.

**Figure 3:**
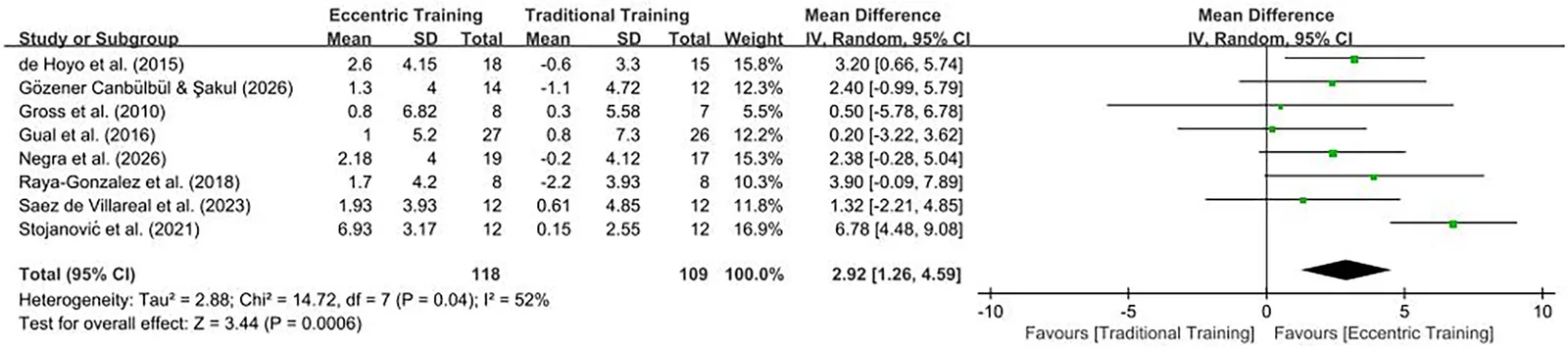
Forest plot for changes in CMJ performance: eccentric training versus conventional training.

In addition, several studies reported other outcome measures related to the effects of eccentric training on athletes’ explosive power. Regarding squat jump (SJ), Gözener Canbülbül and Şakul (2026) demonstrated that the experimental group showed significantly better SJ performance than the control group post-intervention (*p* = 0.002). Similarly, Gross et al. (2010) reported a significant improvement in SJ in the experimental group relative to the control group (*p* = 0.02). For standing long jump (SLJ), Hammami et al. (2021) confirmed that the experimental group significantly outperformed the control group in SLJ performance after training (*p* = 0.015). Negra et al. (2026) further found that the experimental group showed significantly better SLJ performance than the control group (*p* < 0.001). Regarding T-test performance, Hammami et al. (2021) confirmed that the experimental group performed significantly better than the control group after training (*p* = 0.004). Similarly, Hammami et al. (2022) reported a significant improvement in the experimental group compared with the control group (*p* = 0.04). For other outcome measures related to athletes’ explosive power, Komal et al. (2026) showed that eccentric training significantly improved vertical jump performance under different training conditions compared with the control group (*p* < 0.05). Negra et al. (2026) also demonstrated that the experimental group performed significantly better in change of direction (COD) performance than the control group post-intervention (*p* < 0.001). Moreover, Gross et al. (2010) reported that the experimental group exhibited significantly better drop jump (DJ) performance than the control group (*p* < 0.05).

#### Effects of eccentric training on sprint performance

Among the included studies, three studies (de Hoyo et al., 2015; Hammami et al., 2021, 2022) reported data on the 10 m sprint test, involving a total of 98 athletes, as shown in **Figure 4**. One of these studies (Hammami et al., 2021) was a three-arm trial that included an eccentric training group. A random-effects meta-analysis was performed (**Figure 4**); however, given the extremely high heterogeneity (*I²* = 99%), we primarily summarised the results at the individual-study level rather than over-interpreting the pooled estimate. At the individual-study level, de Hoyo et al. (2015) reported a nearly null effect (MD = −0.02, 95% CI: −0.08 to 0.04); the two intervention groups in Hammami et al. (2021) showed inconsistent directions of effect (MD = 0.13 and −0.23, respectively); and Hammami et al. (2022) reported a strong effect favouring the experimental group (MD = −1.20, 95% CI: −1.35 to −1.05). Given the extremely high between-study heterogeneity and the limited number of studies (n = 3), the overall evidence should be interpreted with caution, and future studies with standardised intervention protocols and larger sample sizes are needed to confirm these findings.

**Figure 4:**
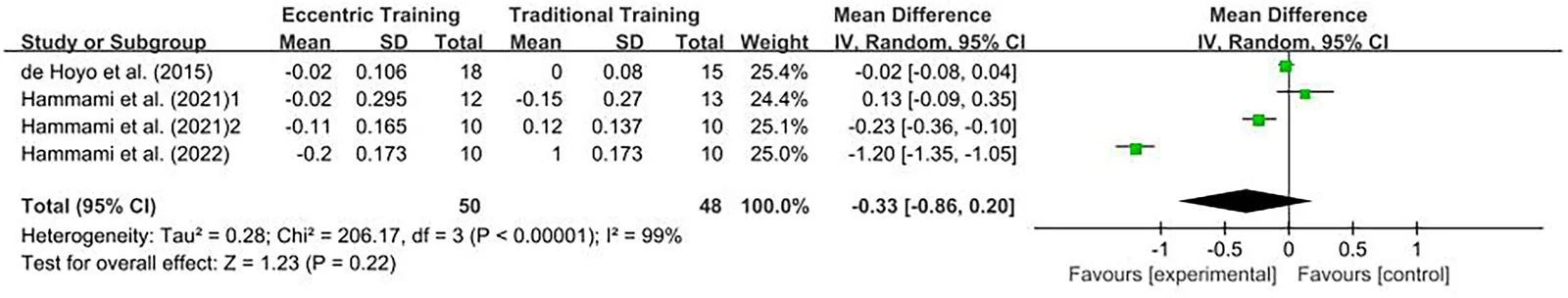
Forest plot for 10 m sprint performance: eccentric training versus conventional training.

Among the included studies, five studies (de Hoyo et al., 2015; Hammami et al., 2021; Negra et al., 2026; Raya-Gonzalez et al., 2018; Stojanović et al., 2021) reported data on the 20 m sprint test in athletes, involving a total of 154 athletes; among them, one study (Hammami et al., 2021) was a three-arm trial that included an eccentric training group. The random-effects meta-analysis showed no statistically significant difference between the eccentric training and conventional training groups in improving 20-m sprint performance (MD = −0.10, 95% CI: −0.21 to 0.02, Z = 1.59, *p* = 0.11). The heterogeneity test indicated substantial between-study heterogeneity (Tau² = 0.01, Chi² = 21.53, *df* = 5, *p* = 0.0006, *I²* = 77%; **Figure 5**). Given the substantial statistical heterogeneity (*I²* = 77%), this pooled effect should be interpreted with caution, despite the use of a random-effects model. To further verify the robustness of the results, a leave-one-out sensitivity analysis was performed. The results showed that after sequentially excluding any single study, the direction of the pooled effect did not reverse (all MDs remained negative), and none of the recalculated 95% CIs crossed zero. This suggests that the overall non-significant result may be influenced by individual studies, and future studies with larger sample sizes and standardised intervention protocols are still needed to confirm these findings.

**Figure 5:**
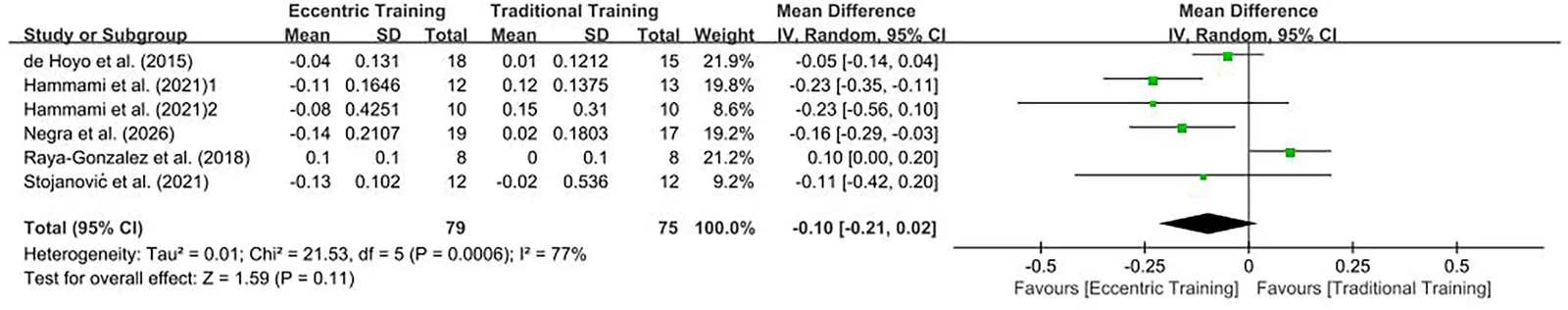
Forest plot for 20 m sprint performance: eccentric training versus conventional training.

Of the included studies, three studies (four comparisons) (Hammami et al., 2021, 2022; Raya-Gonzalez et al., 2018), with one study employing a three-arm design that included an eccentric training group (Hammami et al., 2021), reported data on 30 m sprint performance, involving a total of 81 athletes. The random-effects meta-analysis showed that the eccentric training group did not show a statistically significant improvement in 30-m sprint performance compared with the conventional training group (SMD = −0.42, 95% CI: −1.12 to 0.27, Z = 1.20, *p* = 0.23). Heterogeneity testing revealed moderate between-study heterogeneity (Tau² = 0.28, Chi² = 6.92, df = 3, *p* = 0.07, *I²* = 57%; **Figure 6**). However, given the limited number of included trials, this heterogeneity estimate should be interpreted with caution.

**Figure 6:**
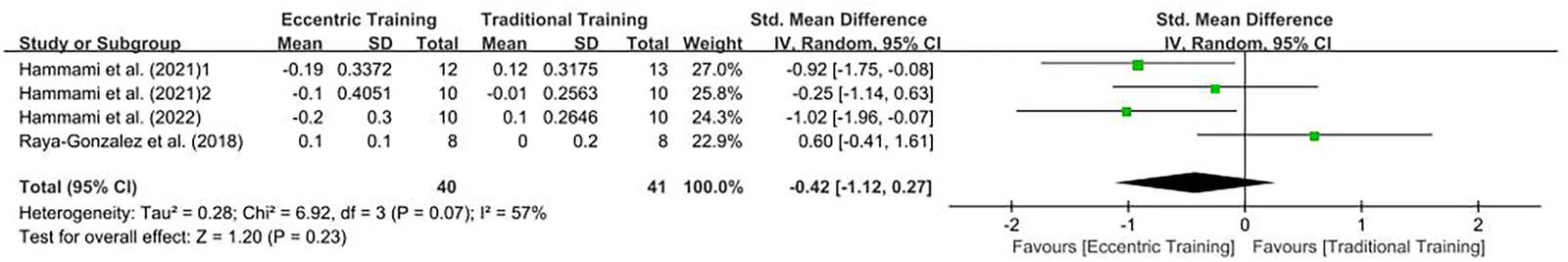
Forest plot for 30 m sprint performance: eccentric training versus conventional training.

In addition, several studies reported the effects of eccentric training on other sprint distances and derived performance indicators. For 15 m sprint performance, Sáez de Villareal et al. (2023) found no significant differences in pre-to-post changes between the eccentric training group and the control group (*p* > 0.05). For 5 m sprint performance, Stojanović et al. (2021) reported a significant difference between the eccentric training group and the control group (*F* = 7.45; *p* = 0.010), indicating that eccentric training effectively improved athletes’ 5 m acceleration ability. Collectively, the effects of eccentric training on sprint performance across different distances remain inconclusive, and the observed effects may be influenced by training modalities. Given the limited number of included studies, these findings warrant further empirical investigation.

### Additional analysis

Subgroup analyses were performed according to participants’ training duration to examine CMJ performance (**Figure 7**). After stratification by training duration, the medium- to long-term training group (≥8 weeks) included five studies, with a pooled MD of 3.41 cm (95% CI: 1.38 to 5.44, Z = 3.30, *p* = 0.001), moderate within-subgroup heterogeneity (*I²* = 61%), and a statistically significant pooled effect. The short-term training group (<8 weeks) included three studies, with a pooled MD of −1.26 cm (95% CI: −4.13 to 1.62, Z = 0.86, *p* = 0.39); the 95% CI crossed zero, indicating no statistically significant difference, with low within-subgroup heterogeneity (*I²* = 26%). The between-subgroup difference was statistically significant (Chi² = 6.77, df = 1, *p* = 0.009, *I²* = 85%), suggesting that training duration significantly moderated the effect of eccentric training on CMJ performance, such that the intervention effect in the medium- to long-term training group (≥8 weeks) was significantly greater than that in the short-term training group (<8 weeks). Furthermore, given the moderate heterogeneity in the ≥8-week subgroup (*I²* = 61%), differences in training protocols (intensity and frequency) and participant characteristics across studies may still exist; therefore, these findings should be interpreted with caution.

**Figure 7:**
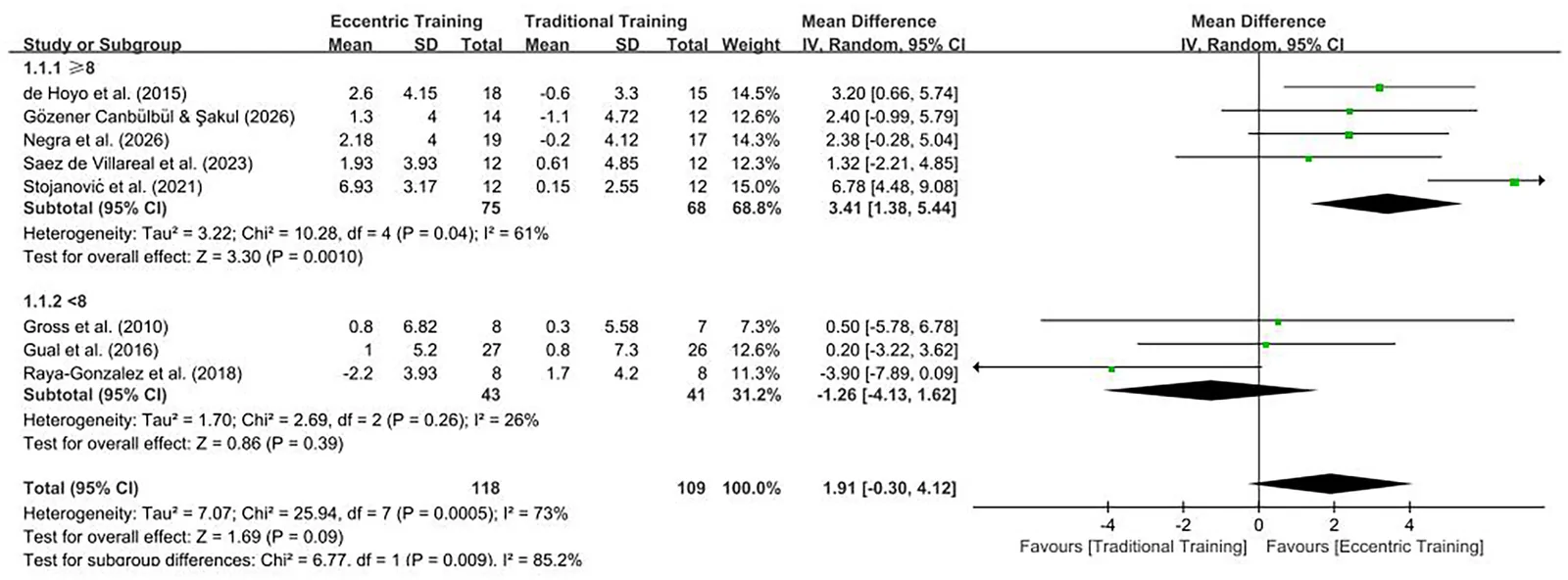
Forest plot for CMJ performance changes by training duration: eccentric versus conventional training.

### Adverse effects

None of the included studies reported any adverse reactions to eccentric training interventions in athletes, such as sports injuries, pain, or other health-related issues.

## Discussion

### Effects of eccentric training on vertical jump performance

This review aimed to compare the effects of eccentric training and conventional training on vertical jump performance in athletes. Because of the number of outcomes available in the included studies, the meta-analysis was restricted to the CMJ in athletes. Therefore, the following conclusions apply primarily to vertical jump performance as represented by the CMJ and should not be directly extrapolated to horizontal displacement tasks such as sprinting. The pooled results suggest that eccentric training may improve CMJ performance in athletes, with a statistically significant difference compared with conventional training. However, the certainty of evidence is limited: few studies were included, each outcome was informed by fewer than 10 studies, publication bias was not assessed, between-study heterogeneity was substantial, and the risk of bias was moderate to high. These findings should therefore be interpreted cautiously as preliminary, hypothesis-generating evidence rather than confirmatory conclusions.

The findings are consistent with Douglas et al. (2017), suggesting that eccentric training may positively affect vertical jump performance in athletes. Compared with previous studies, this review further attempted to explain between-study heterogeneity in terms of intervention duration. In addition, the included eccentric training interventions varied considerably in equipment, movement patterns, eccentric load, contraction velocity, training frequency, intervention period, and concentric coupling methods. This review included only studies explicitly labeled as “eccentric training”; interventions not explicitly labeled as eccentric training, such as Nordic hamstring exercises, were excluded. Owing to data limitations, only intervention duration could be explored in subgroup analyses; other characteristics of eccentric training interventions could not be adequately analyzed. Subgroup analyses suggested that, within 6–24 weeks, eccentric training lasting ≥8 weeks may be more beneficial for CMJ performance than shorter interventions lasting <8 weeks. This finding provides a potential explanation for heterogeneity in previous studies; however, given the small number of studies in the subgroups, substantial heterogeneity, and high risk of bias, it cannot be interpreted as a definitive biomechanical rule or causal relationship. Moreover, this result is based only on intervention duration as a candidate moderator and does not represent the influence of other eccentric training characteristics. In addition, subgroup analyses were based on study-level data and cannot rule out confounding or ecological fallacy. Therefore, intervention duration is more likely a candidate moderator of training effects and still requires validation in prospective studies.

Mechanistically, eccentric training may improve CMJ performance through several pathways. First, eccentric contractions can generate high muscle tension and may promote neural adaptations, such as increased motor unit recruitment, higher firing frequency, and reduced antagonist inhibition (Lepley et al., 2017). Second, eccentric training may induce structural muscle changes, including increased fascicle length, altered pennation angle, and increased tendon stiffness, thereby enhancing stretch-shortening cycle (SSC) efficiency (Gerodimos et al., 2008). In addition, eccentric training may improve force output in the low-velocity, high-force region and enhance eccentric strength and the use of elastic potential energy, all of which may benefit vertical jump performance. It should be noted that both CMJ and horizontal displacement tasks involve eccentric muscle actions; they are not distinguished simply as “eccentric” versus “non-eccentric.” Although the ground contact phase of horizontal displacement tasks is brief, the lower limbs must still rapidly absorb braking forces and reorient the body, imposing substantial eccentric loading whose duration and manifestation differ from those of the CMJ (Marques and Izquierdo, 2014). Thus, using horizontal displacement performance as an outcome has a mechanistic rationale, because eccentric training may affect horizontal displacement performance by improving braking ability, muscle-tendon stiffness, and SSC function, although this transfer may not be identical to that for the CMJ. These tasks also involve factors such as SSC function, muscle-tendon stiffness, rate of force development, ground contact time, and task-specific mechanical demands, all of which may independently influence the transfer of eccentric training adaptations (Xu et al., 2024). Therefore, the vertical–horizontal force direction difference should be considered one dimension for explaining differences in eccentric training transfer, rather than the only or exclusive mechanism. However, these mechanisms are largely based on indirect evidence; this review did not directly measure neuromuscular or biomechanical outcomes, and the mechanistic explanation therefore remains speculative.

At the practical level, these findings may provide preliminary guidance for coaches and athletes when designing eccentric training, particularly because longer interventions (≥8 weeks) may warrant greater attention. However, given the limited certainty of evidence, substantial heterogeneity, and moderate-to-high risk of bias, the current evidence is insufficient to establish definitive training prescriptions or guideline recommendations. Application should be individualized according to athletes’ characteristics, training phase, recovery status, and sport-specific demands. For horizontal displacement tasks such as sprinting, the fact that this review focused on the CMJ should not be taken to imply that such tasks lack eccentric demands or that eccentric training is ineffective; related practical recommendations must await direct evidence. For other jump outcomes, such as the DJ, SLJ, and SJ, the evidence is even less sufficient because few studies were included, and practical recommendations should not be made on this basis. This review has the following limitations: first, the number of included studies was limited, with fewer than 10 studies per outcome, and publication bias was not assessed. Second, between-study heterogeneity was substantial and the risk of bias was moderate to high, reducing the certainty of evidence. Third, owing to the limited number of studies, adequate subgroup analyses by age, competitive level, sex, and other factors could not be performed. Fourth, only the CMJ outcome could be pooled; evidence for other jump outcomes was insufficient. Fifth, task-specific factors such as vertical–horizontal force direction and ground contact time were not systematically evaluated as moderators of eccentric training transfer. Future studies should conduct large-sample, low-risk-of-bias, preregistered randomized controlled trials; harmonize eccentric training protocols, intervention periods, and outcome measurements; perform stratified analyses by age, sex, competitive level, training background, and intervention parameters; investigate neuromuscular mechanisms using electromyography, ultrasound, and force plates; and assess the certainty of evidence using the GRADE system. Until sufficient evidence is available, both mechanistic interpretations and practical promotion of eccentric training should be made cautiously. Current limited evidence suggests that eccentric training may improve CMJ performance in athletes and that interventions lasting ≥8 weeks may be superior to shorter interventions. The current findings support only the possibility that eccentric training may improve CMJ performance; they are insufficient to infer its effects on horizontal displacement tasks such as sprinting, and they do not support reducing the comparison between CMJ to a simple “vertical versus horizontal” or “eccentric versus non-eccentric” distinction. Owing to the small number of studies, substantial heterogeneity, moderate-to-high risk of bias, and the absence of publication bias assessment, these conclusions should be interpreted cautiously and are not yet sufficient to support definitive practical recommendations.

### Effects of eccentric training on linear sprint performance in athletes

Based on the included studies, this review extracted 10 m, 20 m, and 30 m sprint times to compare the effects of eccentric training versus conventional training on linear sprint performance in athletes. The meta-analytic results showed that the pooled effects of eccentric training versus conventional training did not reach statistical significance for any of the three outcomes. It is important to emphasize that these null results should not be interpreted as evidence that eccentric training is ineffective; rather, they indicate that the current pooled evidence is insufficient to support a significant improvement in linear sprint performance with eccentric training. Likewise, the findings should not be interpreted as evidence that linear sprinting lacks eccentric loading or that eccentric training is mechanistically unrelated to sprinting.

The certainty of evidence for these results is limited. Fewer than 10 studies were included for each outcome, and publication bias was not assessed; between-study heterogeneity was substantial, and the risk of bias was moderate to high. Therefore, these results should be regarded as preliminary, hypothesis-generating evidence rather than confirmatory conclusions. This is particularly true for the 10-m sprint, for which between-study heterogeneity was high and only descriptive summary was appropriate; moreover, the direction of findings varied across studies, and the overall effect cannot be inferred by simply counting positive and negative studies.

Compared with the CMJ results, sprint performance did not show significant improvement. This dissociation may be primarily due to task specificity. The CMJ is a bilateral, vertical, preplanned movement that depends on SSC efficiency and vertical force output, whereas sprinting is a single-leg alternating, horizontally directed, cyclical movement with very short ground contact times that relies more on horizontal ground reaction force, horizontal impulse, high-velocity hip extension strength, and intermuscular coordination (Rumpf et al., 2013). These differences represent only one dimension for explaining transfer differences, not the sole or exclusive mechanism. Eccentric training may improve neuromuscular adaptations related to vertical jumping, but if it does not specifically enhance horizontal force output and high-velocity strength, transfer to sprinting may be limited (Salaj and Markovic, 2011). Similarly, this does not mean that linear sprinting lacks eccentric loading or that eccentric training is mechanistically unrelated to sprinting. Although ground contact times in linear sprinting are short, the lower limbs must still rapidly absorb braking forces and reorient the body within an extremely brief period, resulting in substantial eccentric loading (Brady et al., 2024). Thus, including linear sprint performance as an outcome has a mechanistic rationale: eccentric training may influence sprint performance by improving braking ability, muscle–tendon stiffness, and SSC function, although such transfer may not be identical to that for vertical jumping tasks. In addition, because the included studies did not directly measure horizontal force, ground contact time, or electromyographic activity, these mechanisms remain speculative.

Unlike Zhang et al. (2024), this review attempted to pool multiple studies and simultaneously include 10 m, 20 m, and 30 m split times to examine potential differences in the effects of eccentric training across sprint phases. However, this review did not directly measure concentric-dominant push-off or eccentric–concentric coupling efficiency; therefore, it cannot be inferred that eccentric training has differential mechanisms for the acceleration and maximal velocity phases. Likewise, the findings cannot be used to infer that eccentric training is ineffective for the acceleration or maximal velocity phases, nor can they exclude its potential value in specific populations or training contexts. Moreover, these split-time outcomes reflect only linear sprint performance and cannot represent change-of-direction ability or sport-specific explosive actions.

The present findings are inconsistent with those of Bright et al. (2023), in which eccentric training showed more pronounced intervention effects in adolescent athletes. This discrepancy may reflect differences in study populations, training backgrounds, baseline levels, intervention protocols, eccentric training modalities, and outcome measurement methods. Although this review included athletes across a broader age range, the limited number of studies precluded adequate age subgroup analyses; therefore, it cannot be inferred that eccentric training is ineffective at any particular developmental stage, nor can between-study differences be simply attributed to age. Differences in participants’ baseline levels across studies may also influence the results, but this requires validation using individual participant data or large-sample studies.

At the practical level, current evidence is insufficient to recommend eccentric training as a primary or routine intervention for improving linear sprint performance in athletes. If coaches apply eccentric training, it should be regarded as an adjunctive method and individualized according to athletes’ baseline levels, sport-specific demands, training phase, and recovery status, with training responses monitored. For the 10-m sprint, given the high heterogeneity and limited number of included studies, uniform practical recommendations are even less appropriate. The current trial results should not be used to infer that linear sprinting does not require eccentric training or that eccentric training has no value for sprinting; related practical recommendations must await direct evidence.

It should be noted that the outcomes in this review represent linear sprint performance at the level of general physical capacity and cannot be directly equated with sport-specific competition performance or sport-specific training effects. Null results should also not be interpreted as excluding the potential value of eccentric training in specific populations or training contexts. This review has the following limitations: first, the number of included studies was limited, with fewer than 10 studies per outcome, and publication bias was not assessed; second, between-study heterogeneity was substantial and the risk of bias was moderate to high, reducing the certainty of evidence; third, heterogeneity for the 10-m sprint was high, permitting only descriptive analysis, and the results were unstable; fourth, task-specific factors such as vertical–horizontal force direction and ground contact time were not systematically evaluated as moderators of eccentric training transfer.

Future research should conduct large-sample, low-risk-of-bias, preregistered randomized controlled trials; harmonize eccentric training protocols, intervention periods, sprint testing procedures, and outcome measurements; perform stratified analyses by age, sex, competitive level, training background, and intervention parameters; conduct individual participant data meta-analyses when necessary; and combine electromyography, ultrasound, and force plate technologies to investigate the neuromuscular mechanisms by which eccentric training affects linear sprint performance. Current limited evidence does not show that eccentric training significantly improves 10 m, 20 m, or 30 m linear sprint performance in athletes compared with conventional training. Owing to the small number of studies, substantial heterogeneity, moderate-to-high risk of bias, and the absence of publication bias assessment, these conclusions should be interpreted cautiously. Existing evidence is neither sufficient to support eccentric training as a primary means of improving linear sprint performance nor sufficient to conclude that it is ineffective; moreover, it does not support reducing linear sprinting to a simple “vertical versus horizontal” or “eccentric versus non-eccentric” distinction.

### Limitations

This study has the following limitations, which should be considered when interpreting its conclusions.

First, the study was unable to conduct subgroup analyses for key moderators such as sport type, competitive level, characteristics of eccentric training interventions (e.g., equipment, movement patterns, eccentric load, contraction velocity, training frequency, intervention duration, and concentric coupling methods), and training intensity. Although the study planned *a priori* to explore differences in the effects of eccentric training on explosive performance under different conditions according to these variables, the vast majority of included primary studies did not report stratified statistics for each subgroup (e.g., sample size, mean, and standard deviation per subgroup), precluding reliable quantitative pooling. This may cause the overall effect estimates presented in this study to mask true differences among subgroups. For example, gains in explosive performance from eccentric training may be sport-specific (e.g., soccer vs. basketball), and professional and amateur athletes may respond markedly differently to the same training protocol because of differing levels of neuromuscular adaptation. Moreover, different characteristics of eccentric training interventions may lead to different neuromuscular adaptations, but owing to data limitations, this study could not fully distinguish the effects of these characteristics. Therefore, readers should be cautious when extrapolating these conclusions to specific sports or athletes of specific levels.

Second, there are limitations related to participant characteristics. The included studies predominantly involved male athletes; only Gual et al. (2016) used a mixed-sex sample and did not report sex-specific data. This limited the present study’s ability to analyze differences in the effects of eccentric training on explosive performance between male and female athletes.

Third, there are limitations in literature search and coverage. This study searched only six databases indexing Chinese- and English-language publications (Web of Science, PubMed, CNKI, Wanfang, Scopus, and SPORTDiscus) and did not systematically include relevant studies published in other languages. This may have omitted some relevant evidence and may have affected the comprehensive estimation of effect sizes.

Fourth, there are limitations in the methodological quality of the primary studies. According to the PEDro scale, the overall methodological quality of the included studies was low to moderate. Deficiencies in the experimental design of primary studies may have caused pooled effect estimates to be overestimated or underestimated; therefore, the conclusions of this study should be regarded as exploratory evidence.

Fifth, there are limitations related to the insufficient number of included studies. Owing to the limited number of included studies, this study could not conduct subgroup analyses for moderators such as training level, sex, and age, and differences in effects across populations remain to be verified. In addition, only the CMJ outcome and the 10 m, 20 m, and 30 m sprint outcomes could be meta-analyzed. Other explosive performance dimensions (e.g., change of direction and sport-specific explosive actions) could not be pooled because of an insufficient number of studies, and the evidence for these dimensions is even more limited.

Sixth, there are limitations related to heterogeneity in intervention protocols among the included studies. The review included only studies explicitly labeled as “eccentric training” and excluded interventions not explicitly labeled as such, including Nordic hamstring exercises. Even so, the included eccentric training interventions differed substantially in equipment, movement patterns, eccentric load, contraction velocity, frequency, duration, and concentric coupling methods. Some used pure eccentric training, whereas others combined eccentric training with other training forms (combined interventions), making it impossible to clearly attribute effects to eccentric training itself. Such confounding may overestimate or attenuate true effect sizes and limits the strength of inferences regarding the specific effects of eccentric training.

Although random-effects models were used to address heterogeneity among the included studies, deficiencies in the experimental design of primary studies may still lead to overestimation or underestimation of pooled effects. Therefore, the conclusions of this study should be regarded as exploratory evidence, and future studies with higher quality and larger sample sizes are needed to further verify these conclusions. Moreover, the pooled results of this study pertain only to jump performance represented by the CMJ and linear sprint performance represented by the 10 m, 20 m, and 30 m sprints. They cannot be generalized to other explosive performance dimensions, such as change of direction and sport-specific explosive actions, nor do they represent the effects of all eccentric training modalities.

## Conclusions

This systematic review provides preliminary practical guidance for coaches and strength and conditioning professionals regarding eccentric training to improve CMJ performance in athletes. Current evidence suggests that eccentric training may improve CMJ performance in athletes; however, the certainty of evidence is limited, between-study heterogeneity is substantial, and the risk of bias is moderate to high. Therefore, these findings should not be regarded as confirmatory conclusions or a uniform training prescription. Subgroup analyses suggest that intervention duration may be a candidate moderator of CMJ effects, with eccentric training lasting ≥8 weeks potentially superior to shorter interventions lasting <8 weeks. However, this finding is limited by the small number of studies in the subgroups and substantial heterogeneity, requires validation in prospective studies, and should not be interpreted as indicating that the effects of eccentric training are independent of training duration. Coaches are advised to use eccentric training as an adjunct and to individualize it according to athletes’ individual characteristics, training phase, recovery status, and sport-specific demands while monitoring training responses.

For short-distance sprint performance (10 m, 20 m, and 30 m), current evidence did not show significant improvements with eccentric training compared with conventional training, and between-study heterogeneity was high; related conclusions should therefore be interpreted cautiously. However, these findings should not be interpreted as indicating that sprinting does not require eccentric training or that eccentric training has no value for sprinting. Sprinting still involves substantial eccentric loading during ground contact; however, its duration, mechanical purpose, and manifestation differ from those of the CMJ. Moreover, the CMJ and sprinting should not be reduced to a simple dichotomy of “vertical vs. horizontal” or “eccentric vs. non-eccentric.” Therefore, current evidence is insufficient to recommend eccentric training as a primary or routine means of improving linear sprint performance, and it is also insufficient to rule out its potential value in specific populations or training contexts; related practical recommendations must await direct evidence. Furthermore, this review did not pool other explosive performance-related dimensions, such as change of direction, and therefore cannot infer their effects. In addition, evidence on applicability to athletes of different sexes is insufficient. Moreover, this review included only studies explicitly labeled as “eccentric training” and did not include interventions not explicitly labeled as such, such as Nordic hamstring exercises; therefore, the results cannot represent all eccentric training modalities. Future high-quality randomized controlled trials are still needed to determine the optimal eccentric training protocols, dosages, and target populations.

### Practical application

This systematic review provides preliminary practical guidance for coaches and strength and conditioning practitioners regarding the use of eccentric training to improve CMJ performance in athletes. Current evidence suggests that eccentric training may improve CMJ performance; however, given the limited certainty of evidence, substantial between-study heterogeneity, and moderate-to-high risk of bias, these findings should not be regarded as confirmatory conclusions or a uniform training prescription. Subgroup analysis suggested that intervention duration may be a candidate moderator of CMJ effects, with eccentric training lasting ≥8 weeks potentially superior to short-term interventions lasting <8 weeks. However, this finding is limited by the small number of studies in the subgroup and substantial heterogeneity, and it still requires validation in prospective studies; it should not be interpreted as evidence that the effects of eccentric training are independent of training duration. Coaches applying eccentric training are advised to use it as an adjunctive method, individualize it according to athletes’ characteristics, training phase, recovery status, and sport-specific demands, and monitor training responses. For short-distance sprint performance (10 m, 20 m, and 30 m), current evidence does not show significant improvements with eccentric training compared with conventional training, and between-study heterogeneity is substantial; therefore, these conclusions should be interpreted cautiously. This review did not pool analyses for other dimensions of explosive performance, such as change of direction, and thus cannot infer their effects. In addition, evidence regarding applicability across sexes is insufficient, and this review only included studies explicitly labeled as “eccentric training,” excluding interventions such as Nordic hamstring exercise that were not explicitly labeled as such; therefore, the findings cannot represent all eccentric training modalities. Future high-quality randomized controlled trials are still needed to determine the optimal eccentric training protocols, dosage, and target populations.

## Data Availability

The original contributions presented in the study are included in the article/**Supplementary Material**. Further inquiries can be directed to the corresponding authors.
