# Supplementary material for: Effects of eccentric training on athletes’ explosive power: a systematic review and meta-analysis
Source: Front Physiol. 2026 Oct 12;17:1960414. doi: 10.3389/fphys.2026.1960414 (PMC13696304; doi:10.3389/fphys.2026.1960414)
Supplement: Supplementary file 1 [file Table1.docx]

| **S1:Search strategies and results of the included literature** | | |
| --- | --- | --- |
| Datebase | Strategy | Results |
| Wanfang Data | (Eccentric overload training OR Eccentric overload OR Eccentric training OR Eccentric overloading OR Eccentric exercise)AND(Explosive power OR Explosive strength OR Power output OR Rate of force development OR Jump performance OR Sprint performance OR Vertical jump performance)AND(Randomized controlled experiment OR Randomized controlled trial OR Randomized controlled OR Experiment OR Randomized controlled study OR Randomized experiment) | 44 |
| SPORTDiscus | ((AB (athlete∗) OR AB (player∗) OR AB (player))AND(AB (Randomized controlled trials) OR AB (Randomized controlled trials) OR AB (Randomized controlled) OR AB (experiment) OR AB (Randomized Experiment) OR AB (rondom*))AND([AB (Explosive power) OR AB ((Explosive strength) OR AB (power output) OR AB (Rate of force development) OR AB (Jump performance) OR AB (Sprint performance) OR AB (plyometric performance)](https://research.ebsco.com/search/results?db=aph,agr,awh,amed,cul,8gh&expanders=concept&limiters=None&searchMode=all&sort=relevance&sqId=sq:791fad4d-fa2d-45da-bdc7-f056a3134bdb&userDirectAction=true))AND(AB (eccentric training) OR AB (flywheel overload training) OR AB (eccentric overload) OR AB (flywheel exercise) OR AB (eccentric overload performanc) OR AB (flywheel sport) OR AB (YOYO sport) OR AB (YOYO exercis) OR AB (YOYO training) OR AB (eccentric training)) | 718 |
| PubMed | (((eccentric training) OR (((((((((flywheel overload training[Title/Abstract]) OR (eccentric overload[Title/Abstract])) OR (flywheel exercise[Title/Abstract])) OR (eccentric overload performanc[Title/Abstract])) OR (flywheel sport[Title/Abstract])) OR (YOYO sport[Title/Abstract])) OR (YOYO exercis[Title/Abstract])) OR (YOYO training[Title/Abstract])) OR (eccentric training[Title/Abstract]))) AND (((((((Explosive power) OR (Explosive strength)) OR (Power output)) OR (Rate of force development)) OR (Jump performance)) OR (Sprint performance)) OR (plyometric performance))) AND ((Randomized controlled trials[Title/Abstract]) OR (((((Randomized controlled trials[Title/Abstract]) OR (Randomized controlled[Title/Abstract])) OR (experiment[Title/Abstract])) OR (Randomized Experiment[Title/Abstract])) OR (rondom*[Title/Abstract]))) | 529 |
| Web of Science | (((eccentric training) OR (((((((((flywheel overload training[Title/Abstract]) OR (eccentric overload[Title/Abstract])) OR (flywheel exercise[Title/Abstract])) OR (eccentric overload performanc[Title/Abstract])) OR (flywheel sport[Title/Abstract])) OR (YOYO sport[Title/Abstract])) OR (YOYO exercis[Title/Abstract])) OR (YOYO training[Title/Abstract])) OR (eccentric training[Title/Abstract]))) AND (((((((Explosive power) OR (Explosive strength)) OR (Power output)) OR (Rate of force development)) OR (Jump performance)) OR (Sprint performance)) OR (plyometric performance))) AND ((Randomized controlled trials[Title/Abstract]) OR (((((Randomized controlled trials[Title/Abstract]) OR (Randomized controlled[Title/Abstract])) OR (experiment[Title/Abstract])) OR (Randomized Experiment[Title/Abstract])) OR (rondom*[Title/Abstract]))) | 757 |
| CNKI | ("eccentric overload training" OR "eccentric overload" OR "eccentric training" OR "eccentric overloading" OR "flywheel" OR "eccentric exercise")AND("explosive power" OR "explosive strength" OR "power output" OR "rate of force development" OR "jump performance" OR "sprint performance" OR "vertical jump performance") AND("randomized controlled experiment" OR "randomized controlled trial" OR "randomized controlled" OR "experiment" OR "randomized controlled study" OR "randomized experiment") | 16 |
| Scopus | ((TITLE-ABS-KEY (RCT) OR TITLE-ABS-KEY (quasi-experimental) OR TITLE-ABS-KEY (intervention study) OR TITLE-ABS-KEY (longitudinal study))) AND ((TITLE-ABS-KEY (randomized controlled trial) OR TITLE-ABS-KEY (experimental study) OR TITLE-ABS-KEY (quasi-experimental study) OR TITLE-ABS-KEY (longitudinal study) OR TITLE-ABS-KEY (intervention study))) AND ((TITLE-ABS-KEY (Explosive power) OR TITLE-ABS-KEY (Explosive strength) OR TITLE-ABS-KEY (Power output) OR TITLE-ABS-KEY (Rate of force development) OR TITLE-ABS-KEY (Jump performance ) OR TITLE-ABS-KEY (Sprint performance) OR TITLE-ABS-KEY (plyometric performance) OR TITLE-ABS-KEY (power) OR TITLE-ABS-KEY (jump) OR TITLE-ABS-KEY (sprint) OR TITLE-ABS-KEY (performance))) AND ((TITLE-ABS-KEY (eccentric training) OR TITLE-ABS-KEY (flywheel overload training) OR TITLE-ABS-KEY (eccentric overload) OR TITLE-ABS-KEY (flywheel exercise) OR TITLE-ABS-KEY (eccentric overload performanc) OR TITLE-ABS-KEY (flywheel sport) OR TITLE-ABS-KEY (YOYO sport) OR TITLE-ABS-KEY (YOYO exercis) OR TITLE-ABS-KEY (YOYO training) OR TITLE-ABS-KEY (eccentric training))) | 503 |
| Total |  | 2567 |
